# Pollination Strategies of *Eranthis stellata* (Ranunculaceae), a Spring Ephemeral with Elaborate Petals

**DOI:** 10.3390/biology14070804

**Published:** 2025-07-03

**Authors:** Jiudong Zhang, Jie Sui, Lin Wang, Xianhua Tian, Xiaohui Zhang, Jing Xu, Tianpeng Gao

**Affiliations:** 1 School of Biological and Environmental Engineering, Xi’an University, Xi’an 710065, China; zhangjiudong029@163.com (J.Z.); jing.xu@xawl.edu.cn (J.X.); 2 College of Life Sciences, Shaanxi Normal University, Xi’an 710119, China; jidelikeyiyi@163.com (J.S.); wangmumu234@163.com (L.W.); tianxh@163.com (X.T.); xhzhang@snnu.edu.cn (X.Z.); 3 Engineering Center for Pollution Control and Ecological Restoration in Mining of Gansu Province, Lanzhou City University, Lanzhou 730070, China

**Keywords:** deception, false nectary, petal function, pollination strategy

## Abstract

Spring ephemerals have different pollination strategies to avoid the rareness and/or low activity of insect pollinators caused by low temperature in early spring. However, limited research has been conducted on the effects of elaborate petals and the pseudonectaries on petals on pollinator attraction. We examined the role of the elaborate petals and the pseudonectaries in pollinator attractions of the spring ephemeral *Eranthis stellata* (Ranunculaceae) using a combination of observational and experimental approaches. The results indicated that the color contrast created by the yellow pseudonectaries that did not secrete nectar on the white petals, was more attractive to both bees and flies as nectar guides, but only bees with relatively longer tongues could access nectar at the base of the petal tubes. Also, the pseudonectaries were food deception for fly visitors. Food deception as a mechanism to increase the efficiency of pollination has not been reported for Ranunculaceae or other basal eudicots.

## Simple Summary

*Eranthis stellata* has dichogamous flowers in which the stamens started shedding pollen continuously for three to four days. Floral nectar secretion began when the sepals opened and lasted until the petals were shed. A total of six insect species were observed visiting *E. stellata*, including two bee species and four fly species. *Platycheirus scutatus* had the highest visiting frequency. The visiting frequency of the bees decreased significantly after petals, stamens, and pseudonectaries were removed, or after the pseudonectaries were coated with starch. The yellow pseudonectaries that did not secrete nectar on the white petals, might be attractive to both bees and flies as nectar guides, but only bees with relatively longer tongues could access nectar at the base of the petal tubes. Also, the pseudonectaries were food deception for fly visitors.

## 1. Introduction

Spring ephemerals are understory perennial herbs that emerge shortly after snow melt and complete their aboveground growth, including fruit production, within two months before canopy closure [1,2,3]. Plants flowering in early spring encounter cool and highly variable weather conditions. Low temperature is probably the most limiting factor for insect-mediated pollination and pollinator availability, including the rareness and/or low activity of insect pollinators [4,5]. There are various flowering strategies of spring ephemerals in response to the low insect pollinator availability to increase seed set. A “sit-and-wait” hypothesis suggests that the plants could increase flowering longevity to ensure the success of sexual reproduction [6]. The heliotropic movement of flowers is another strategy, effectively increasing the floral temperature and attracting insect pollinators without any cost of heat or nectar production [7]. Another hypothesis is the competition strategy, which considers that the time and period of flowering have been responsive to pollinator activity and intensity of competition for pollinator acquisition [8,9]. However, Rathcke and Lacey [10] considered that traits other than flowering times may lessen potential competitive effects, though attractiveness of the floral morphological traits in spring ephemerals was largely ignored.

Attracting animal pollinators is essential to the sexual reproductive success of the great majority of angiosperm species [11]. The angiosperm flower, particularly the corolla, attracts pollinators through the elaborate architecture of flowers and/or the floral organs. Several features of the corolla have been considered in terms of their effects on pollinator attraction, including nectar guides, which consist of converging lines, dots, or a marking around the corolla aperture [12,13], and pseudonectaries, which may function both as nectar guides and to deceive pollinators [14,15]. Most animal-pollinated flowers combine two or more attractive elements, including color, scent, pollen, and nectar [16,17,18].

*Eranthis stellata* Maxim., known as a winter aconite species (*Eranthis* Salisb., Ranunculaceae), is a spring ephemeral [19] distributed in broadleaved deciduous forests in Jilin and Liaoning Provinces, China, as well as in North Korea and the Russian Far East [20]. The flower of *E. stellata* is solitary and white, with small petals or honey leaves [21]. Each of the petals is bilabiate, with a long stalk. The tubular part of the petal has nectar at the bottom, and the lower lip is bifid, with a yellow drop-like protuberance from the ventral base of each lobe. The yellow drop-like protuberance forms a central ring-shaped marking, which stands out against the backdrop of the white sepals and petals. The architecture of the flower, especially the petal, of *E. stellata* is different from that of other spring ephemerals. This leads us to the question, what is the role of such elaborate petals in the process of pollination? Endress and Matthews [22] considered the yellow drop-like protuberances on the petal lobes of *Eranthis* as pseudonectaries. Moreover, Weber [23] suggested that the pseudonectary acts as a nectar guide in *Nigella arvensis* Linn (Ranunculaceae)., which has a petal morphology like that of *E*. *stellata*. We tested these hypotheses by manipulating the presence and appearance of the pseudonectaries and testing the effects of this manipulation on the behavior of floral visitors (flies and bees).

## 2. Materials and Methods

### 2.1. Morphological and Flowering Dynamics

Field observations were carried out from April 1 to 30, 2020–2023, at Hamagou, Zuo’an, Dongchang District (41°43′19″ N, 126°01′01″ E, alt. 375 m), Tonghua, Jilin Province, China. The individuals of *Eranthis stellata* occupied an area of 1000 × 150 m at the site, and two 10 × 10 m sample plots were observed, which were approximately 500 m apart. During the budding phase, thirty flower buds at a similar developmental stage were labeled each year. After the flower opened, each flower was observed for 9 to 12 days (total 45–62 h) and the flower longevity, the maturity status of the stamens and carpels, and the withering order of each floral organ were documented. Twenty additional fully opened flowers were collected and fixed immediately in FAA (formalin to acetic acid to alcohol = 5:5:90, volume ratio), and the length of the petal and petal tube were measured in 2020. Fifteen additional flower buds were randomly selected and bagged each year from 2022 to 2023, and the nectar volume from all of the petals for each flower was measured with a 0.5 μL microcapillary tube once per day at 4 pm. After the nectar was measured, the flowers were bagged again to exclude visitors, and nectar measurements were repeated 9 to 12 days in a row.

The morphology and histology of the petals were observed with a scanning electron microscope (SEM) and a light microscope. For SEM observations, fixed materials were dehydrated in an ethanol and isoamyl acetate series, treated with critical point drying using CO_2_ as a transitional fluid, vacuum evaporated, and observed and photographed with a Hitachi S-3400 SEM. The materials used for histological observation were dehydrated in an ethanol series, infiltrated with xylene, and embedded in paraffin wax. The embedded material was sectioned at 10 μm thickness and stained with safranin and fast green. Sections were observed and photographed using a Leica DM5000B microscope equipped with a Leica DFC490 camera.

### 2.2. Pollen Viability and Stigma Receptivity Tests

Pollen viability was tested using the MTT method (1% 3-(4,5-dimethylthiazol-2-yl)-2,5-diphenyl-tetrazolium bromide or thiazolyl blue) in 5% sucrose [24]. Ten flower buds were randomly selected and bagged prior to blooming in each year from 2021 to 2022. A few pollen gains per anther were collected at intervals of 8 h for 9 to 12 days after anther dehiscence and treated with MTT. For each test, we placed a sample of pollen in a 5–10 μL droplet of the reagent on a slide, intensively mixed the sample and reagent, and let the sample dry. Then we repeated the procedure by adding additional pollen and reagent. We then added a droplet of glycerin to the stained and dried sample and examined the slide under a Nikon E200 microscope (ocular × objective = 10 × 10). The pollen grain was considered viable if it had peroxidase activity by turning dark violet-purple-brown, whereas it was considered not viable if it remained yellow.

Stigma receptivity was monitored via peroxidase activity at different stages of anthesis using hydrogen peroxide [25]. Sixty additional green flower buds were marked and bagged each year from 2021 to 2022. The receptivity of five stigmas from each of the five flowers was tested at the following stages: when the flower buds changed from green to white, flowers began to open, and the anthers in the outermost whorl dehisced. We observed and counted the number of flower anthers opening three times a day at 8 a.m., 12 p.m., and 5 p.m. After all anthers of a flower dehisced, the receptivity of five stigmas from each of the five flowers was tested at intervals of 8 h to determine the duration of receptivity. Stigma receptivity lasted for nine to twelve days. To test for receptivity, we put a droplet of hydrogen peroxide reaction solution (the volume ratio of 1% benzidine, 3% hydrogen peroxide, and water is 4:11:22) onto the stigmas. A blue color developed in 2–5 min if peroxidases were present; stigmas that stained blue were scored as receptive.

### 2.3. Visitors and Their Visiting Behavior and Frequency

Thirty additional flowers were selected, and the visitors and their visiting behaviors were observed and recorded each year. Preliminary observations in 2020 indicated that no insect visitors were present on the flower of *E*. *stellata* on cloudy, snowy, or rainy days and from 4 pm of the first day to 9 am of the next day during sunny days; therefore, observations were conducted from 9 am to 4 pm every day during anthesis from 2021 to 2023. During observations, the following information was recorded on every visit for any visitor: (1) visiting behavior of each visitor on the flower(s), including: Where do the visitors land on the flowers, sepals, or other parts of flowers? Do the visitors lick the pseudonectaries? Do the visitors insert their mouth parts into the petal tube? Do the visitors contact the stamens and stigma?; (2) visiting times, the number of times an individual flower was visited by each insect species within an hour, which was used to calculate the visiting frequency; and (3) the duration of visit, which was timed with a stopwatch.

Ten samples for each insect species were collected in transparent wide-mouthed bottles. Five of the samples were fixed with 50% alcohol for identification, and the remaining five were dried at room temperature in the lab and coated with gold–palladium using a sputter coater to observe the pollen-carrying position and amount of pollen with SEM. The pollen morphology of *E. stellata* from the unopened anthers was also observed with SEM to confirm that the pollen on the bodies of visitors was indeed the pollen of *E*. *stellata*. Measurements were taken on freshly killed (using ethyl acetate) insects. The length of the mouthparts was measured in different ways for different insect taxa. For the hymenopteran group, the length measured was the distance between the tip of the stretched glossa (using fine forceps to gently stretch it) and the basal extreme of the prementum, as recommended by Harder [26]. For the dipteran group, the proboscis length was measured on the fully extended proboscis, from the head to the labellum tip.

### 2.4. Petal and Pseudonectary Manipulations

To test the roles of the petals, pseudonectaries, and stamens in pollination, thirty additional flowers were randomly selected each year from 2021 to 2023 and divided into five groups: (1) control, (2) petals removed, (3) pseudonectaries removed, (4) pseudonectaries coated with starch, and (5) stamens removed. The following data were observed and recorded: visiting behavior, visiting frequency, and visiting duration for each bee and fly species. The effects of flower manipulation on visitation frequency of both bees and flies were analyzed by one-way ANOVA followed by Duncan’s multiple contrasts tests. All statistical tests were implemented using SPSS (v. 19).

## 3. Results

### 3.1. Floral Morphology and Flowering Dynamics

**Figure 1:**
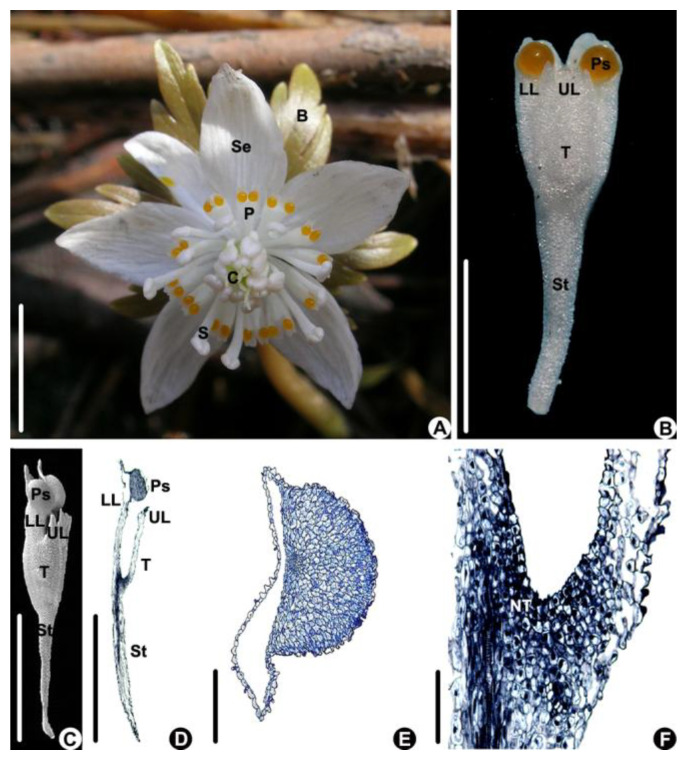
The flower and petal of *Eranthis stellata*. (**A**) Flower of *E. stellata* (above view). (**B**–**F**) Petal morphology and anatomy. (**B**,**C**) Petal morphology. (**B**) Fresh petal (above view). (**C**) A petal under SEM (side view). (**D**–**F**) Longitudinal sections of a petal. (**D**) An entire petal. (**E**) Magnified part of a pseudonectary, showing that it consists of non-secretory cells. (**F**) Magnified part of the lower part of the petal tube, showing the secretive tissue. (Bar: (**A**–**D**) = 1 cm, (**E**,**F**) =100 μm). B: bract, C: carpel, LL: lower lip of petal, NT: nectary tissue, P: petal, Ps: pseudonectary, S: stamen, Se: sepal, St: petal stalk, T: petal tube, UL: upper lip of petal.

Flower of *Eranthis stellata* are solitary; consist of 5–6 sepals, 9–16 petals, 18–40 stamens, and 5–12 carpels; and have foliaceous bracts beneath the flower (n = 20, Figure 1A). The tepals are white and elliptical (Figure 1A). The tepals are white; obcuneate; 5.66 ± 0.67 mm in length, which is about 1/3 of the length of the sepal; and 1.81 ± 0.33 mm in width (Figure 1B); and there is a stalk, which is about 1/2 of the length of the petal, at the lower part and a tube at the upper part (Figure 1B,C). The tube is bilobiate at the upper part and has a longer lower lip and shorter upper lip (Figure 1B–D). The lower lip is bilobed and has a drop-like protuberance at the base of each lobe on the ventral side (Figure 1B–D). The protuberance consists of non-secretory cells characterized by having smaller nuclei and a light-stained cytoplasm (Figure 1E). The petal tube is about 1.78 ± 0.01 mm long (Figure 1B,C) and has nectar-producing tissue at its base characterized by small cells with larger nuclei and a densely stained cytoplasm (Figure 1D,F).

**Figure 2:**
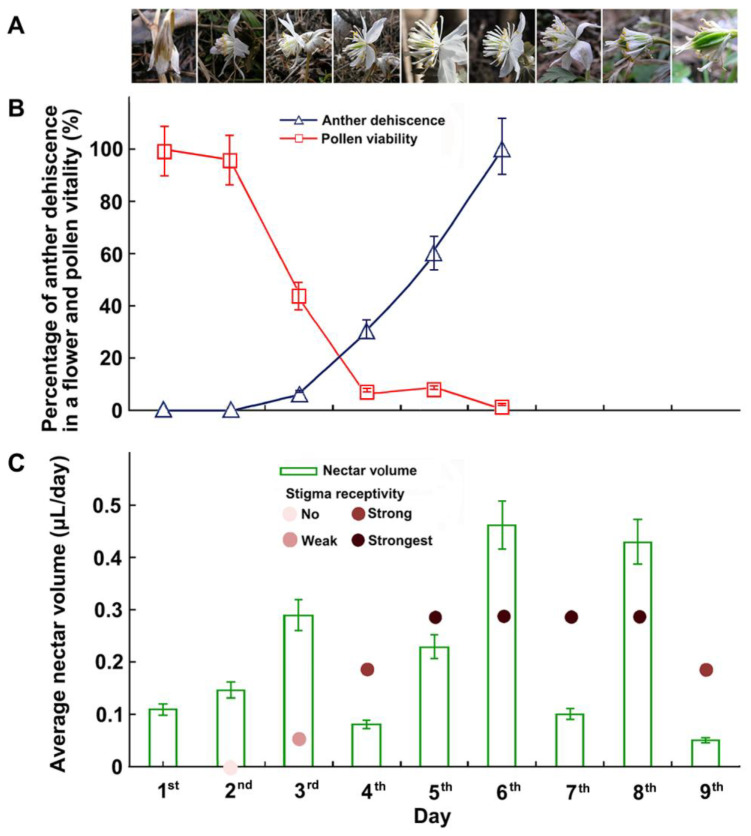
Flowering dynamics and changes in anther dehiscence, pollen viability, stigma receptivity, and nectar volume during the flowering of *E. stellata*. (**A**) Flowering dynamics of an individual flower. Day 1: flower bud began to open. Day 2: sepals completely opened. Day 3: petals and stamens flared, filaments elongated, anthers of outer stamens dehisced. Day 5: flowers fully opened; anthers were as high as stigmas. Day 7: flower was pollinated, sepals reflexed, petals and stamens furled. Day 8: sepals fell off, young fruit enlarged. Day 9: petals fell off, stamens were falling off. (**B**) Changes in percentage of dehiscent anthers in a flower, pollen viability on different days during flowering. Pollen viability began to decrease after approximately 48 h and was reduced to 50% or less after approximately 80 h (error bars show the standard error). (**C**) Changes in nectar volume and stigma receptivity on different days during flowering.

*Eranthis stellata* emerged from beneath the snow under the plant litter at the beginning of April. The stems of *E. stellata* sprouted quickly and extended aboveground in the shape of an “n”. The flower buds extended quickly after the ground was exposed, followed by the bracts and opening sepals. After one day, the sepals were fully opened and the petals, stamens, and carpels were completely visible. When the flowers began to open, the gynoecia were slightly higher than the androecia. The stamens gradually spread outward, filaments extending, and the outer stamens began to shed pollen, and these processes slowly moved inwards. After the flowers were pollinated, the sepals were the first floral organs to wither and then fall off, followed by the stamens, and the petals were shed about one to two days after the stamens completely fell off (Figure 2A).

*Eranthis stellata* has dichogamous flowers in which the stamens started shedding pollen continuously for three to four days, beginning 24 to 36 h after flowering began. Pollen viability began to decrease approximately 48 h after initial pollen spreading and decreased to 50% or less after approximately 80 h (Figure 2B). Stigmas became receptive 28 to 32 h after the flower opened. Stigma receptivity peaked 36 to 72 h after the flower opened and ceased nine days after the onset of flowering (Figure 2C).

Floral nectar secretion began when the sepals opened and lasted until the petals were shed (Figure 2C). The average nectar amount per flower was 0.14 µL (0.03 µL–0.24 µL) per day.

### 3.2. Visitors and Their Visiting Behavior

**Figure 3:**
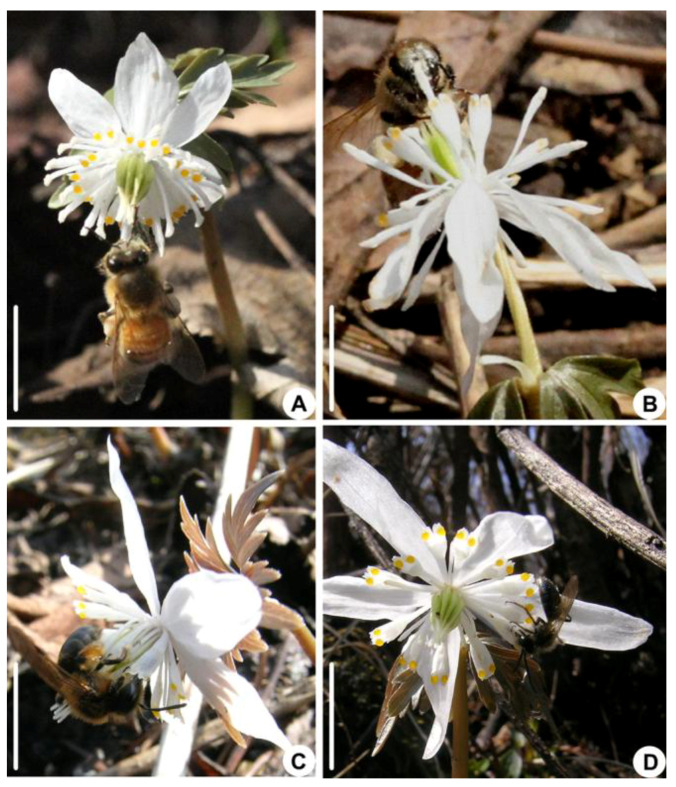
Bee pollinators of *E. stellata*. (**A**,**B**) Asian honey bees (*Apis cerana*) sucking nectar in the petal by using their forelegs to grasp the stigmas. (**C**,**D**) Melittid bees (*Melitta taishanensis*) foraging nectar from the petal. (Bar: (**A**–**D**) = 1cm).

**Figure 4:**
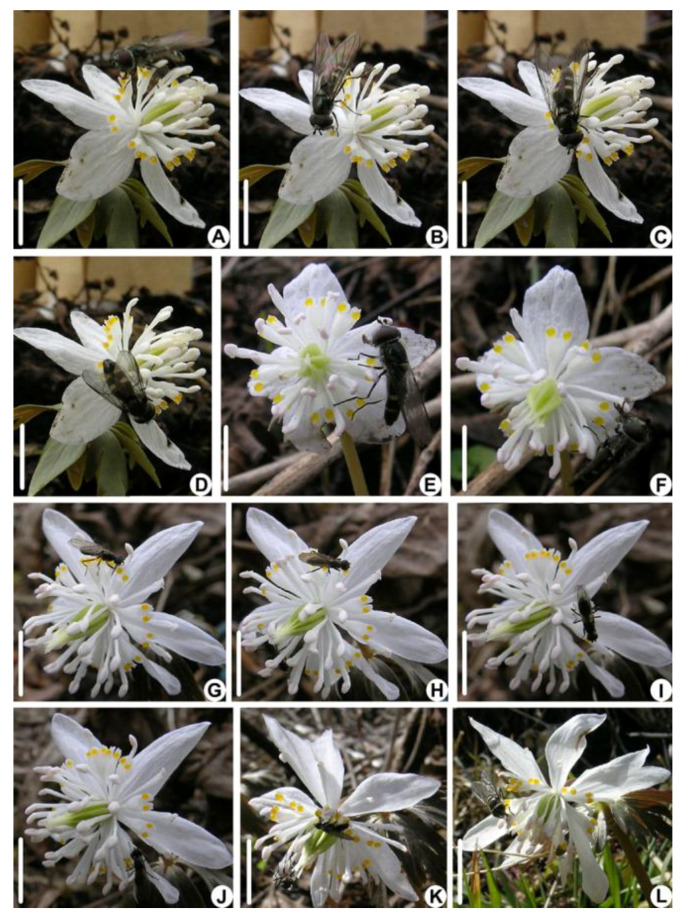
Fly pollinators of *E. stellata*. (**A**–**E**) Series of photos showing a hoverfly (*Platycherius scutatus*) walking on the petals and licking the pseudonectaries. (**F**) A hoverfly foraging for pollen by holding the stigmas. (**G**–**K**) Series of photos showing an anthomyiid fly (Anthomyiidae sp.) walking on the petals and licking the pseudonectaries. (**L**) An anthomyiid fly foraging for pollen. (Bar: (**A**–**I**) = 1 cm).

A total of six insect species were observed visiting *E. stellata*, including two bee species (Asian honey bee: *Apis cerana cerana*, Apidae, Figure 3A,B, and melittid bee: *Melitta taishanensis*, Melittidae, Figure 3C,D) and four fly species. The fly species include two species of hoverfly (*Platycheirus scutatus*, Syrphidae; Figure 4A–F), anthomyiid fly (Anthomyiidae sp.; Figure 4G–L), Milesiinae sp., and fruit fly (*Drosophila* sp., Drosophilidae). The longhorn bee (*Eucera longicornis*, Apidae) was observed visiting only once during the study period. Bees carried more pollen grains than flies (Table 1). Based on the SEM observation, the bees mainly carried *E. stellata* pollen grains on the head, abdomen, thorax, and legs; the thorax and legs of the Asian honey bee and the abdomen of the melittid bee carried more pollen grains. Among the fly species, only *Platycheirus scutatus* carried pollen on the head, legs, and abdomen, while *Milesiinae* sp. carried pollen only on their legs. Other species of flies carried few pollen grains (Table 1).

**Figure A1:**
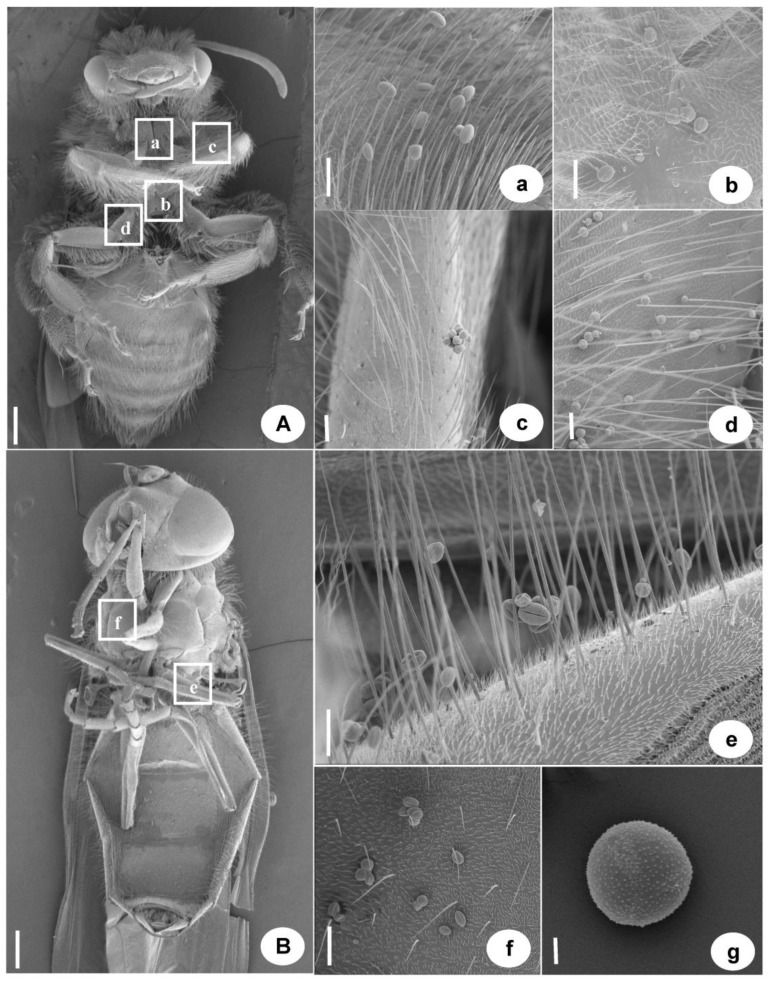
Two Main Pollinating Insects in Turmeric by Scanning Electron Microscopy of Eranthis stellata. (**A**) Chinese honeybee (a–d. pollen carried by Chinese honeybees), a. Pollen carried in the chest, b. Carrying pollen between the first and second pairs of appendages, c. The pollen carried by the first pair of appendages, d. The pollen carried by the second pair of appendages. (**B**) syrphid (e,f. Pollen carried by aphid eating flies), e. Pollen carried by appendages, f. Pollen carried in the chest, g. The pollen of Eranthis stellata. (**A**,**B**) Bar. = 1 mm, a–f Bar. = 100 μm, g Bar. = 10 μm.

The visiting behavior of the two bee species was similar. Immediately after landing on *E. stellata*, the bees (both have chewing–lapping mouthparts) stood over and grasped the stigmas and the stamens and then inserted their mouthpart into a petal tube between two pseudonectaries to forage for nectar (Figure 3A–D). The length of the bees’ proboscis was longer than the length of the nectar tube (Table 1), and the bees could suck nectar. While foraging, bees were observed touching the stigmas with their thorax and the anthers with their legs. After finishing foraging in the first petal, the bees moved to the other petals one by one to suck nectar from the same flower, and in this process, the insect could touch the stigmas and the anthers (Figure A1). The Asian honey bees spent 30.35 ± 19.37 s (Table 1) foraging nectar from three to five petals on a single flower and 7.59 ± 4.84 s on average to forage nectar from a petal. The melittid bees spent 58.26 ± 39.56 s (Table 1) foraging nectar from 9 to 16 petals on a single flower and 4.66 ± 3.16 s on average to forage nectar from a petal. No pollen-collecting behavior was observed for the bee species.

**Figure A2:**
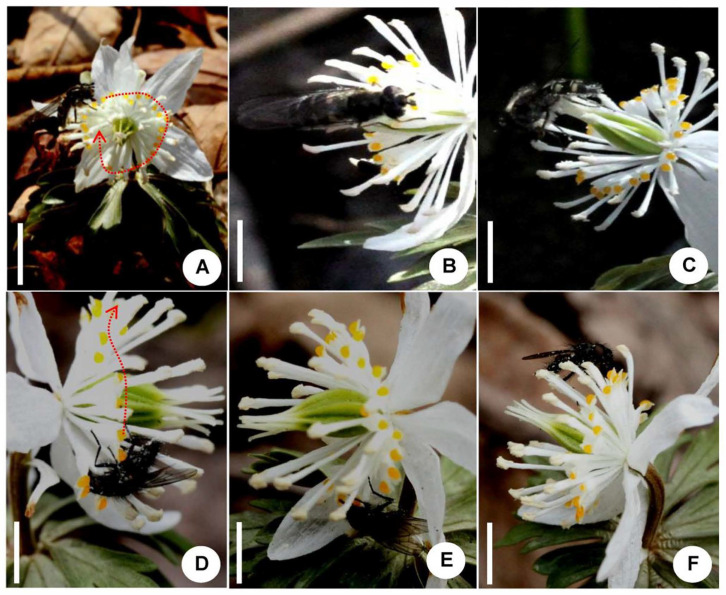
Fly like insects’ flower visiting behavior in Eranthis stellata. (**A**–**C**). Platycherius scutatus crawls and licks the false nectaries on the petals, and feeds on pollen and licks the false nectaries with the stigma as a fulcrum. (**D**–**F**). Insects of the family Drosophidae insert their mouthparts into the petals, crawl along the petals, and lick the false nectaries. The red dashed line represents the route of flies on flowers. Bar. = 1 cm.

The visiting behavior of the four fly species was similar. After landing on the flower, flies used their spongy mouthparts to lick the pseudonectaries on four to six petals (Figure 4A–D,G–J). Then, some flies attempted to lick the nectar but failed because their mouthpart was shorter than the petal tube (Table 1) or rarely succeeded in inserting their mouthpart into the petal tube (Figure 4E). During this process, the flies usually walked on the sepals and petals, rarely touching the stamens and seldom touching the stigmas (Figure 4A–D,G–J). Then, the flies moved on to the stamens, crawling over the flower and licking up pollen grains from the anthers (Figure A2). During this process, the head, abdomen, and legs of the flies had the potential to touch the anthers and stigmas (Figure 4F,K,L and Figure A1). The flies spent more time, 45.29 ± 17.9 s, on the petals than on stamens, 26.09 ± 8.44 s (Table 1). *Platycheirus scutatus* had the highest visiting frequency (Table 1).

### 3.3. Petal, Stamen, and Pseudonectary Manipulations

**Figure 5:**
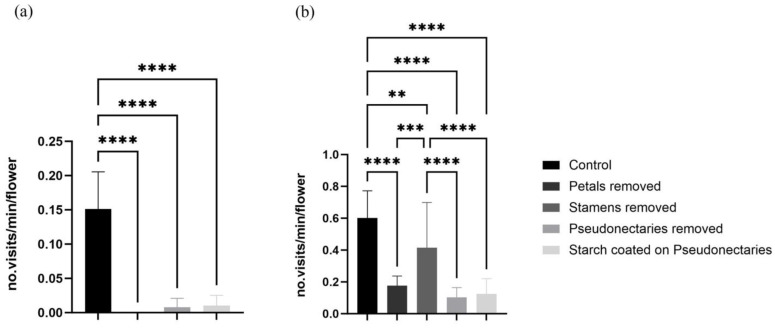
Comparison of average visiting frequency of flies and bees in minutes after stamen removal, petal removal, pseudonectary removal, or coating of pseudonectaries with starch. (**a**) Visiting frequency of bees. (**b**) Visiting frequency of flies. Unpaired *t*-tests were used for statistical analysis (** *p* < 0.01, *** *p* < 0.001, **** *p* < 0.0001).

The visiting frequency of the bees decreased significantly after petals, stamens, or pseudonectaries were removed, or after the pseudonectaries were coated with starch (Figure 5a). Bees were observed only visiting the flowers seven, zero, and three times during the observation periods in 2021, 2022, and 2023, respectively. The bees foraged nectar occasionally from the petal tube and stayed for only three to five seconds on a flower after the pseudonectaries were removed or coated. The visiting frequency of the flies was also significantly reduced after the petals or pseudonectaries were removed, or after the pseudonectaries were coated (Figure 5b), but the visiting frequency was not significantly reduced after the stamens were removed (Figure 5b).

## 4. Discussion

Endress and Matthews [22] indicated that the yellow drop-like protuberances on the petal lobes are the pseudonectaries in *Eranthis*, but this assumption had not been tested. Based on the histological observations of the present study, the cells in the protuberances had small nuclei and light-stained cytoplasm, which do not match the characteristics of the nectary tissue according to the definition of nectary tissue [27]. The cells in the inner bottom of the petal tubes had densely stained cytoplasm and large nuclei, and thus were nectary cells.

### 4.1. Pollinators of Eranthis Stellata

Our combined data identified the pollinators of *E. stellata* as five out of the six observed species. The two bee species (Asian honey bee and melittid bee) and three fly species (both hoverflies and the anthomyiid fly) are considered the pollinators for *E. stellata*, indicating a generalized pollination system. Although the fruit fly displayed similar visiting behavior as the other three fly species, it was not considered a pollinator because it did not carry pollen grains. Our findings agreed with those presented by Kudo [28] and Jannathan [29], which revealed that the flies and a small number of bees are the main pollinators in early spring. Flies are considered major floral visitors and reliable pollinators in early spring under cool temperature conditions when other pollinators are inactive [30,31], because flies are not affected by low temperature [28].

It is generally considered that bees are more effective pollinators than flies [32,33]. Compared to fly visitors, bees consistently contacted the stigmas and stamens while collecting nectar and carried more pollen. In contrast, flies only touched the stamens or stigmas when they foraged for pollen and carried fewer pollen grains than bee visitors. Therefore, we consider bees to be more effective pollinators of *E. stellata* than flies.

### 4.2. Multiple Roles of Petals in Pollination

It is well known that petals are often the most attractive floral organ in angiosperm flowers. Our observations infer that the elaborate petals, especially the pseudonectaries and the nectar produced at the bottom of the petal tube, of *E. stellata* play multiple roles in pollination. The first role is that the pseudonectaries on the petals can be food deceptive to fly visitors. The food deceptive species imitate a range of floral attractants, such as floral shape, color, and scent, that are associated as an edible reward by the pollinators [14,34,35]. In the present study, results show that the pseudonectaries acted as food deception for the fly visitors based on the following three facts. The first fact is that the pseudonectaries may mimic the nectar drops on petals in both color and shape. The second fact is that fly visitors first lick the pseudonectaries, which consist of non-secretory cells, after landing on the flowers, and then forage for pollen grains from the anthers; the flies spend more time licking the pseudonectaries than licking anthers. The third fact is that after the petals were removed, which also included the removal of pseudonectaries, the visiting frequency of flies was significantly reduced. This apparent food deception might act to prolong the duration of the visiting time of fly visitors, thus increasing their effectiveness as pollinators. Food deception mostly occurs in Orchidaceae species [36,37,38] but has also been sporadically reported in other Ranunculaceae or other basal eudicots [39,40].

The second role is the color contrast produced by the yellow pseudonectaries, which are arranged in a conspicuous ring, standing out against the white petals, sepals, and stamens. This color pattern showed the role of petals for the following reasons: (1) the frequency of visits by bees decreased significantly with the removal of pseudonectaries or petals and when pseudonectaries were coated in starch, (2) petal removal had a similar effect on the visitation frequency of flies, and (3) during flowering, the plants are surrounded by snow cover and insects may not easily distinguish flowers from the snow-covered background. Indeed, removing petals or pseudonectaries, or coating the yellow pseudonectaries with white starch, may eliminate the color contrast, possibly reducing the ability of insects to detect the flowers. This color contrast might serve to attract visitors from a long distance away, especially bees, which can easily recognize the yellowness and are able to target floral guides [41,42]. The bees no longer visited the flower after the petals were removed, and the visitation of bees decreased after the removal or coating of pseudonectaries, showing the strong attraction of pseudonectaries, which highlights the color contrast to the bees because the pseudonectaries serve as advertising to the bee that nectar is available. The visiting frequency of the fly species also decreased when the petals were removed, indicating that the color contrast also effectively attracts flies.

The pseudonectary of *Nigella arvensis* Linn. (Ranunculaceae) shares similar petal morphology to *E. stellata*, which has been considered a nectar guide [23]. In *E. stellata*, bees can insert their mouthparts directly and accurately into the opening of the petal tube to forage for nectar as soon as they land on the flower, and a bee forages for nectar in three to five petals of a flower within a very short period during a visit. That might indicate that pseudonectaries probably do not indicate that nectar is available, but rather where nectar is available. Thus, we suggest that the third role of pseudonectaries might also help distract visitors from the pollen on the anthers. We were unable to collect enough samples to concretely hypothesize that the pseudonectaries might act as a nectar guide through the removal or coating of pseudonectaries because bees visited far less frequently after the pseudonectaries were removed or coated. However, from the few observed instances of bee visiting, we found that the bees stayed for only a few seconds and occasionally foraged the nectar from the petal after they landed on a flower that had removed or coated pseudonectaries. This could indicate that the bees could not find the signal that the nectar was available in the flower, so they did not need to stay on the flower for long.

More generally, the petals of *E*. *stellata* might also serve to attract pollinators via the production of nectar, and hidden nectar could increase foraging difficulty and prolong the handling time on the flowers for nectar-sucking pollinators, thus facilitating more effective pollination, which is considered to act as the reward for pollinators [43,44]. In fact, it seemed that the duration of visiting time for bee species was very short in *E*. *stellata*. For *E. stellata*, nectar is produced at the base of the petal in an enclosed tube. Among the pollinators observed in this study, only bees had mouthparts sufficiently long enough to access the nectar. Therefore, the petals might also serve to prevent the removal of nectar resources by flies, which appear to be less effective pollinators of *E*. *stellata* than bees. Therefore, we consider that the fifth role of the petals might be to restrict flies from accessing nectar to avoid the nectar being foraged by less effective pollinators; this may be why the nectar was hidden in the petal tube.

### 4.3. Pollination Strategies of Eranthis Stellata

The pollination strategy may be diverse for different spring ephemerals. Diverse pollination strategies can even occur in different closely related genera because each genus has a different evolutionary history, such as the differences among Anemone, Adonis, and *Eranthis* in Ranunculaceae [45]. Although there have been some hypotheses on the pollination strategies of spring ephemerals [6,7,8], the function of the specific floral traits has been largely ignored [10], and there has been limited research on the mechanisms that affect pollinator visitation other than studies of flowering times. There has been limited research on how spring ephemerals increase their attractiveness through elaborate petals and pseudonectaries on the petals. Based on the present study, we assume that the pollination strategy of *E. stellata* is to increase its floral attractiveness to bee and fly pollinators through the elaborate petals that have multiple functions, including color contrast, a nectar guide, and a nectar reward for bees, and food deception and a pollen reward for flies. These pollination strategies allow *E. stellata* to attract a variety of pollinators, thus avoiding pollinator limitation due to low temperatures in the early spring.

## 5. Conclusions

Five out of the six observed species, two bee species (Asian honey bee and melittid bee) and three fly species (both hoverflies and the anthomyiid fly) are considered the pollinators for *E. stellata*, indicating a generalized pollination system. The pseudonectaries and the nectar produced at the bottom of the petal tube of *E. stellata* play multiple roles in pollination. We assumed that the pollination strategy of *E. stellata* is to increase its floral attractiveness to bee and fly pollinators through the elaborate petals that have multiple functions, including color contrast, a nectar guide, and a nectar reward for bees, and food deception and a pollen reward for flies. These pollination strategies allow *E. stellata* to attract a variety of pollinators, thus avoiding pollinator limitation due to low temperatures in the early spring. Future research should focus on the long-term monitoring of pollination dynamics, particularly exploring the broader ecological consequences of bees and flies on native early spring plant pollinator networks.

## Figures and Tables

**Table 1:** Visiting frequency, visiting duration, foraging, and pollen-carrying position and amount for different visitors.

Visitor	Length of Proboscis (mm)	Foraging	Pollen-Carrying Position and Amount				Visiting Frequency (Times /Flower/min) (Mean ± s. e., Range)	Average Time Spent on Stamens (Second)	Average Time Spent on Petals (Second)
			Head	Thorax	Abdomen	Leg			
Diptera									
Hoverfly (*Platycheirus scutatus*)	0.53 ± 0.042	Pollen	+	-	++	+	0.166 ± 0.02 (0.08–0.24)	23.10 ± 8.74	54.45 ± 15.82
Fly (*Milesiinae* sp.)	0.46 ± 0.021	Pollen	-	-	-	+++	0.11 ± 0.02 (0.47–0.25)	26.06 ± 9.45	42.43 ± 19.51
Fruit fly (*Drosophila* sp.)	-	Pollen	-	-	-	-	0.07 ± 0.01 (0.03–0.12)	23.03 ± 7.35	39.33 ± 18.93
Hymenoptera									
Asian honey bee (*Apis cerana cerana*)	4.03 ± 0.25	Nectar	-	+++	+++	+++	0.10 ± 0.02 (0.06–0.16)	0	30.35 ± 19.37
Melittid bee (*Melitta taishanensis*)	6.32 ± 0.26	Nectar	+	+	+++	++	0.08 ± 0.01 (0.05–0.11)	0	58.26 ± 39.56

Note. “-” carrying no pollen grain, “+” carrying less than five pollen grains, “++” carrying six to ten pollen grains, “+++” carrying more than eleven pollen grains.

## Data Availability

The raw data are available from the first author.
